# Supplementary material for: A fault-intrusion-tolerant system and deadline-aware algorithm for scheduling scientific workflow in the cloud
Source: PeerJ Comput Sci. 2021 Nov 2;7:e747. doi: 10.7717/peerj-cs.747 (PMC8576554; doi:10.7717/peerj-cs.747)
Supplement: Supplemental Information 6 [file peerj-cs-07-747-s006.zip › Myproject1.1/docs/org/cloudbus/cloudsim/examples/power/Constants.html]

Constants


JavaScript is disabled on your browser.


- Overview
- Package
- Class
- Use
- Tree
- Deprecated
- Index
- Help

- Prev Class
- Next Class

- Frames
- No Frames

- All Classes

- Summary:
- Nested |
- Field |
- Constr |
- Method

- Detail:
- Field |
- Constr |
- Method


org.cloudbus.cloudsim.examples.power

## Class Constants

- java.lang.Object
- - org.cloudbus.cloudsim.examples.power.Constants

- ---

    

  ```
  public class Constants
  extends java.lang.Object
  ```

  If you are using any algorithms, policies or workload included in the power package, please cite
  the following paper:
  Anton Beloglazov, and Rajkumar Buyya, "Optimal Online Deterministic Algorithms and Adaptive
  Heuristics for Energy and Performance Efficient Dynamic Consolidation of Virtual Machines in
  Cloud Data Centers", Concurrency and Computation: Practice and Experience (CCPE), Volume 24,
  Issue 13, Pages: 1397-1420, John Wiley & Sons, Ltd, New York, USA, 2012

  Since:
  :   Jan 6, 2012

- - ### Field Summary

    Fields

    | Modifier and Type | Field and Description |
    | --- | --- |
    | `static int` | `CLOUDLET_LENGTH` |
    | `static int` | `CLOUDLET_PES` |
    | `static boolean` | `ENABLE_OUTPUT` |
    | `static int` | `HOST_BW` |
    | `static int[]` | `HOST_MIPS` |
    | `static int[]` | `HOST_PES` |
    | `static PowerModel[]` | `HOST_POWER` |
    | `static int[]` | `HOST_RAM` |
    | `static int` | `HOST_STORAGE` |
    | `static int` | `HOST_TYPES` |
    | `static boolean` | `OUTPUT_CSV` |
    | `static double` | `SCHEDULING_INTERVAL` |
    | `static double` | `SIMULATION_LIMIT` |
    | `static int` | `VM_BW` |
    | `static int[]` | `VM_MIPS` |
    | `static int[]` | `VM_PES` |
    | `static int[]` | `VM_RAM` |
    | `static int` | `VM_SIZE` |
    | `static int` | `VM_TYPES` |
  - ### Constructor Summary

    Constructors

    Constructor and Description

    `Constants()`
  - ### Method Summary

    - ### Methods inherited from class java.lang.Object

      `clone, equals, finalize, getClass, hashCode, notify, notifyAll, toString, wait, wait, wait`

- - ### Field Detail


    - #### ENABLE\_OUTPUT

      ```
      public static final boolean ENABLE_OUTPUT
      ```

      See Also:
      :   Constant Field Values


    - #### OUTPUT\_CSV

      ```
      public static final boolean OUTPUT_CSV
      ```

      See Also:
      :   Constant Field Values


    - #### SCHEDULING\_INTERVAL

      ```
      public static final double SCHEDULING_INTERVAL
      ```

      See Also:
      :   Constant Field Values


    - #### SIMULATION\_LIMIT

      ```
      public static final double SIMULATION_LIMIT
      ```

      See Also:
      :   Constant Field Values


    - #### CLOUDLET\_LENGTH

      ```
      public static final int CLOUDLET_LENGTH
      ```

      See Also:
      :   Constant Field Values


    - #### CLOUDLET\_PES

      ```
      public static final int CLOUDLET_PES
      ```

      See Also:
      :   Constant Field Values


    - #### VM\_TYPES

      ```
      public static final int VM_TYPES
      ```

      See Also:
      :   Constant Field Values


    - #### VM\_MIPS

      ```
      public static final int[] VM_MIPS
      ```


    - #### VM\_PES

      ```
      public static final int[] VM_PES
      ```


    - #### VM\_RAM

      ```
      public static final int[] VM_RAM
      ```


    - #### VM\_BW

      ```
      public static final int VM_BW
      ```

      See Also:
      :   Constant Field Values


    - #### VM\_SIZE

      ```
      public static final int VM_SIZE
      ```

      See Also:
      :   Constant Field Values


    - #### HOST\_TYPES

      ```
      public static final int HOST_TYPES
      ```

      See Also:
      :   Constant Field Values


    - #### HOST\_MIPS

      ```
      public static final int[] HOST_MIPS
      ```


    - #### HOST\_PES

      ```
      public static final int[] HOST_PES
      ```


    - #### HOST\_RAM

      ```
      public static final int[] HOST_RAM
      ```


    - #### HOST\_BW

      ```
      public static final int HOST_BW
      ```

      See Also:
      :   Constant Field Values


    - #### HOST\_STORAGE

      ```
      public static final int HOST_STORAGE
      ```

      See Also:
      :   Constant Field Values


    - #### HOST\_POWER

      ```
      public static final PowerModel[] HOST_POWER
      ```
  - ### Constructor Detail


    - #### Constants

      ```
      public Constants()
      ```


- Overview
- Package
- Class
- Use
- Tree
- Deprecated
- Index
- Help

- Prev Class
- Next Class

- Frames
- No Frames

- All Classes

- Summary:
- Nested |
- Field |
- Constr |
- Method

- Detail:
- Field |
- Constr |
- Method
